# Using Social Media While Waiting in Pain: A Clinical 12-Week Longitudinal Pilot Study

**DOI:** 10.2196/resprot.4621

**Published:** 2015-08-07

**Authors:** Mark Merolli, Kathleen Gray, Fernando Martin-Sanchez, Steven Mantopoulos, Malcolm Hogg

**Affiliations:** ^1^ Health and Biomedical Informatics Centre The University of Melbourne Melbourne Australia; ^2^ Pain Management Services The Royal Melbourne Hospital Melbourne Australia; ^3^ Department of Anaesthesia and Pain Management The Royal Melbourne Hospital Melbourne Australia

**Keywords:** chronic pain, chronic disease, participatory health, patient-reported outcomes, self-management, social media, therapeutic affordances, pilot study

## Abstract

**Background:**

Chronic pain places an enormous burden on health care systems. Multidisciplinary pain management services are well documented as an effective means to improve patient outcomes. However, waiting lists to access these services are long and outcomes deteriorate. Innovative solutions such as social media are gaining attention as a way to decrease this burden and improve outcomes. It is a challenge to design research that demonstrates whether social media are acceptable to patients and clinically effective.

**Objective:**

The aim was to conduct a longitudinal pilot study to understand what aspects of research design are key to the success of running a larger-scale study of social media use in the clinical management of chronic pain.

**Methods:**

A 12-week study examined social media use by patients on the waiting list for the Royal Melbourne Hospital Pain Management Service. Selected social media resources were suggested for use by patients waiting for an appointment at the clinic. Patients filled out measures for pain interference and pain self-efficacy before and after the study. Follow-up was conducted at monthly intervals via telephone semistructured interviews to discuss engagement and garner individual perceptions towards social media use. A social media-use instrument was also administered as part of the after-study questionnaire.

**Results:**

Targeted recruitment refined 235 patient referrals to 138 (58.7%) suitable potential participants. Contact was made with 84 out of 138 (60.9%) patients. After a further exclusion of 54 out of 84 (64%) patients for various reasons, this left 30 out of 84 (36%) patients fitting the inclusion criteria and interested in study participation. A final study cohort of 17 out of 30 (57%) was obtained. Demographics of the 17 patients were mixed. Low back pain was the primary condition reported as leading to chronic pain. Semistructured interviews collected data from 16 out of 17 (94%) patients who started the trial, and at final follow-up 9 out of 17 (53%) patients completed questionnaires. Low specificity of the resources to one’s condition and time poorness may have been barriers to engagement.

**Conclusions:**

Results suggest that with refinements, this study design can be implemented successfully when conducting a larger social media study. At present, comment cannot be made on what effect using social media can have on patients on hospital waiting lists, nor whether those who use social media while waiting in pain achieve better outcomes from eventual participation in a chronic pain program. Long-term follow-up should be included in future studies to answer this. Future research should focus on multicenter randomized controlled trials, involving patients in the intervention design for improved participation and outcomes and for evidence to be sound.

## Introduction

Chronic pain is one facet of chronic disease placing a significant burden on health care systems and individuals alike, through stigma, personal suffering, loss of income, and isolation. It is estimated to affect approximately 1 in 5 Australians and to cost the economy AUD $34 billion a year [1-4]. One consequence has been increased waiting list times to access clinic-based pain management services (up to 6 months). This correlates to poorer patient-reported outcomes (PROs) for quality of life (QOL), and for psychological and physical well-being [2,5].

Specialized multidisciplinary team approaches and pain management programs are well-established forms of management [1] for the primary reasons of cost, social interaction, and sensations of validation among attendees. There has been a strong national endeavor across pain services to monitor patient outcomes to ensure that treatments provided actually result in improved function and QOL for patients [6]. Regardless, the health care system struggles to provide timely access to such services. Chronic pain self-management support is, therefore, crucial.

Barlow and colleagues describe self-management as the individual’s ability to manage the symptoms and treatment, as well as the physical and social consequences of lifestyle changes linked to living with a chronic condition [7]. Various self-management interventions have been described, for example the Stanford model of chronic disease self-management program, acceptance and commitment therapy, and cognitive behavioral therapy (CBT) [1]. From a broader health care system point of view, benefits of effective self-management strategies can include decreased medical consultations, hospitalizations, and imaging investigations. From a patient point of view, benefits include decreased use of analgesic and days off work, as well as improved QOL, mood, self-efficacy, and empowerment, to name a few [1,8,9].

Innovative and cost-effective strategies are recommended to alleviate the pressing need for self-management support [2,10]. Web-based resources are one area gaining attention [1,5,10]. These may be implemented as stand-alone interventions, as adjuncts to traditional care, or as tools to bridge the waiting time between obtaining a referral from a general practitioner and receiving a place in a pain clinic program [11]. Recent examples in Australia include the following: painHEALTH from Western Australia [12] and the Pain Management Network from New South Wales [13]. However, it is a challenge to demonstrate that such resources are clinically effective and accepted by patients [11].

The success of Web-based resources may lie in their ability to provide individuals with a means to tailor management to their own needs, as well as a channel for support outside of traditional clinic hours [1,11]. This is a distinct advantage over offline chronic pain management; there is the potential to reach large, diverse populations at relatively low cost and provide geographically unrestrained access to resources. Hence, their appeal for reaching people with chronic pain (PWCP) in remote areas, those with changing schedules, stigmatized and isolated individuals, and those who have mobility issues [11].

Social media—sometimes referred to as Health 2.0 tools—provide an innovative approach to Web-based resources. Unlike general Web-based interventions, social media are characterized by more highly evolved platforms that allow enhanced user engagement and autonomy, as well as greater social functionality and interaction [14,15]. This ultimately allows patients to better appraise their own individual situation. In social cognitive theory this is called self-efficacy [1] and it can be enhanced by peer modeling and support, things that social media may enable. Patients may thus become more empowered and engaged in collaborative self-management [16-18].

Studies have examined social media use in chronic pain [19-21]. However, none have examined the utility of social media for PWCP on waiting lists for specialist pain services. Nor have any studies involved patients in the study design process, with a focus on their perceptions and motivations for using different social media [22]. Results from studies conducted to date are not yet sufficient to inform design or implementation of social media interventions in clinical settings [23]. Greater emphasis is needed on conceptual research frameworks within a participatory health research paradigm for social media to become useful tools in chronic pain self-management [24].

Therefore, the aim of this pilot study was to understand what aspects of research design are key to the success of running a larger-scale study of social media use in the clinical management of chronic pain.

## Methods

### Overview

The methodology of this pilot study aligns with previous research into the therapeutic affordances of social media in PWCP [25-27]. This approach recognizes that each patient has individualized needs, building on the principles of participatory health care [28].

The setting chosen for the study was the Royal Melbourne Hospital—Pain Management Services (RMH-PMS). The Royal Melbourne Hospital (RMH) is a large publicly funded hospital in Melbourne, Australia. It offers one of the most comprehensive outpatient pain management services in the country. RMH-PMS has between 900 and 1100 outpatient referrals per year. At any one time, there are from 200 to 300 PWCP on the waiting list, and they have a wait time of 6 to 8 months for a first appointment. RMH-PMS provides an ideal context in which to test a social media intervention.

### Ethics

The Human Research Ethics Committees of Melbourne Health and the University of Melbourne approved this study (ID No. 2014.043). During the review of the project, the ethics committee specifically considered the following areas of study design:

1. The sample: comments were made about representativeness and selection of the sample, as well as the impact using social media may have on expectations of patients for management of their condition.

2. Social media resources: feedback centered on anticipated outcomes and what impact social media would have on participants’ conditions. Queries related to the appropriateness of the resources, who would be responsible for the content, and monitoring the resources.

3. Logistics and staff allocation: this covered staff and time required to recruit, to manage participant numbers during the study, to collect data, and to conduct follow-up.

### Targeted Patients and Recruitment

The sample pool consisted of patients with chronic pain—pain of greater than 3-months duration [29,30]—on the RMH-PMS outpatient waiting list. Sampling was sequential and inclusion criteria were applied (see Table 1). Exclusion criteria were also applied, including the following: a change in priority status (ie, immediate intervention required) or discharge from the waiting list.

**Table 1:** Study inclusion criteria.

Inclusion criteria	Comments
Competent in English (reading and writing)	
Regular Internet access and competent usage abilities based on a preexisting, validated model [31]	An Internet-literate cohort was sought because Internet users were the primary point of reference for this study.
Willing to register with Gmail and Facebook (if they didn’t already have accounts) and be bound to each site’s terms and conditions	
Not currently undertaking any online intervention to manage their pain	
Not currently using chronic pain-related social media resources for management of this condition	We defined this as follows: being a member of a pain support group online and/or regularly reading blogs, watching videos, and contributing to forums about pain (this did not exclude general/personal social media use).
Medically appropriate (based on physical/psychological status, cognitive function, visual/hearing function)	This was determined by clinical study investigators.

A clinically relevant sample was sought. Desired cohort size for this study was estimated based on the following factors: participant numbers used in pilot studies within a similar domain [32-38], the number of referrals to the RMH-PMS program within a 3- to 4-month period, and the number of patients managed in the pain service at any particular time. Based on a combination of these factors, the aim was to enroll approximately 20 patients.

Potential patients classified as *priority 2* were sourced from the RMH-PMS Direct Access Unit referrals database. *Priority 2* includes patients receiving support via a primary care practitioner while waiting for a consult at RMH-PMS. Referrals are received from general practitioners in the local area, community health centers, other medical specialists, and internal referrals from RMH. Each referral was reviewed for preliminary suitability by two of the study’s investigators who met nine times between April and September 2014. Potentially suitable participants were flagged based on matching information recorded in their referral with study inclusion/exclusion criteria (ie, English language ability, current interventions/treatments, any significant conditions requiring treatment noted in the history). Recruitment was conducted via phone calls from July to September 2014. The people reached were advised of typical waiting list times and introduced to this study as a self-management strategy available in the meantime. The phone transcript can be found in Multimedia Appendix 1.

Interested patients were emailed with a link to the study website and a unique identifier. The link led to SurveyMonkey—an online survey site—where the study information, consent form, and pretest questionnaire were hosted. After 1 week, follow-up phone calls were made to discuss the study, determine suitability, confirm Internet competence, and to ensure the prestudy questionnaire was submitted to confirm registration in the study. Once registered, contact was made to discuss final details and educational material was emailed that contained links to the suggested social media resources and instructional videos—filmed by the study’s primary investigator—to act as an introduction to the resources and provide instructional information for using the resources. The educational material can be found in Multimedia Appendix 2.

Patients who were ineligible to participate in the study were provided with information about online resources (ie, painHEALTH [12]) if they desired for their future reference.

### The Intervention

Each enrolled patient completed the intervention over 12 weeks and because recruitment was staggered, some commenced at different points in time. The entire intervention period ran from July 24 to December 5, 2014. Waiting list time is typically between 6 and 8 months with specialist preclinic pain education provided close to the medical admission appointment. Therefore, treatment was not delayed by completing this study. No incentives were offered to participate. It was also explained that the study was a pilot study and that participant feedback may help to shape future consideration of social media resources for the RMH-PMS.

Patients were informed that the intervention consisted of using the suggested social media-based pain management resources in an unrestrained fashion over the allotted time period. Patients were asked to interact with these at their own pace. They were given the autonomy to be selective as to which they interacted with and how. Of interest was the impact that use might have on their condition and on their understanding about pain management, and finally, to know how they interacted with the resources—this would be examined during monthly semistructured interviews that will be described further.

The social media resources incorporated into this study included a large chronic pain support community on Facebook, a selection of chronic pain blogs, and pain management YouTube videos filmed by painHEALTH. Further detail about these resources and their selection for this study can be found in Multimedia Appendix 3. These particular platforms were suggested based on social media used by PWCP from a global online survey investigating social media use in chronic pain self-management. Also, platforms were selected based on each platform’s ability to foster various therapeutic affordances that appear conducive to positively impacting health effects in chronic pain [26,27]. The resources were reviewed and agreed on by all study investigators from both the Health and Biomedical Informatics Centre at the University and RMH-PMS.

### Outcome Measurement

Pre- and postintervention data were collected, as well as data collected at monthly intervals during the study. To start, participants completed a questionnaire on SurveyMonkey that amalgamated demographic information, chronic pain status, and patient-reported outcome measures (PROMs)—pain interference and pain self-efficacy—into one survey (see Multimedia Appendix 4). Patient-reported outcomes (PROs) provide insight into the patient’s perception of the impact that interventions have on their health [18].

Pain interference (PI) is an example of a regularly examined standardized outcome that measures the burden on an individual across a wide range of health-related quality-of-life (HRQOL) measures [39,40]. PI was measured using 16 items from the Patient-Reported Outcomes Measurement Information System—Pain Interference (PROMIS-PI) item bank and included one item from the pain behavior item bank to measure pain severity. Unlike common legacy outcome measures used to measure chronic pain, the PROMIS-PI item bank demonstrates good reliability and validity across a variety of chronic diseases, including chronic pain, and shows strong correlations to other common outcome measures, allowing findings from this study to be compared and generalized in the future [39,40].

Pain self-efficacy (PSE), or confidence in one’s ability to perform certain tasks in the face of pain, was measured using the 10-item Pain Self-Efficacy Questionnaire (PSEQ) [41]. Social cognitive theory underlying PSE was discussed briefly in the introduction [1]. Based on social media’s ability to foster peer modeling and support, it was logical to include the PSEQ as a primary measure. The PSEQ has frequently been cited as a standardized outcome measure in chronic pain management. Accordingly, *efficacy* determines the effort and persistence an individual will apply when faced with hurdles and adverse experiences. Like PROMIS-PI, the PSEQ provides a means to overcome inconsistency and generalizability among previous existing legacy measures covering a range of general behaviors and activities, and directly contextualizes self-efficacy in regard to living with pain [41,42]. Similar to the argument made for the use of PROMIS-PI, the PSEQ is favored over legacy self-efficacy measures because it more effectively overcomes the variability in measurement presented by other instruments.

At the end of the study, patients were guided to a second SurveyMonkey questionnaire that amalgamated all PROMs (PROMIS-PI and PSEQ) and a social media-use instrument into one survey (see Multimedia Appendix 5). The social media-use instrument sought to examine which resources were used, the amount used, features of each used, positive/negative aspects of using the resources, and perceptions toward various therapeutic affordances. The same line of questioning was used for each resource—Facebook page, blogs, and YouTube videos. Questions about therapeutic affordances were phrased to better understand the degree to which each affordance is present and impacts PROs. Five therapeutic affordances of social media, refined through a global online survey of PWCP [26], were examined through 15 statements, each consisting of three exploratory components. These measured the following: (1) self-presentation—preferences regarding one’s identity, (2) connection—using social media to connect with others, (3) exploration—guidance toward useful information, (4) narration—sharing experiences of chronic pain, and finally (5) adaptation—motivation, frequency, and type of use.

At monthly intervals during the intervention period, patients were also contacted on the phone by the primary investigator to complete a brief 10- to 15-minute, semistructured interview (see Multimedia Appendix 6). Semistructured interviews are well suited to small-scale studies with small participant numbers, and their utility lies in their ability to collect rich qualitative data that can be analyzed in a variety of ways to supplement pre- and posttest survey data [43,44]. Data collection gave people a chance to discuss participation and study progress, but more importantly offered patients the opportunity to help shape social media use in future studies and interventions by collecting information regarding social media use and perceptions toward the five therapeutic affordances free of coercion. These phone calls were not medical consultations and patients were advised of this. Any queries of a medical nature were flagged for follow-up by a member of the clinical team.

### Data Analysis

Major study design procedures and the study process are the focus of the results described. Brief descriptive statistics of pre- and posttest survey data are also presented. Pertinent to the study was the examination of barriers to engagement with the resources and intervention. To explore these, thematic analysis was employed using a grounded theory *inductive* phenomenological approach on the semistructured interview data to uncover any emergent themes.

*Deductive* coding of semistructured interviews was also conducted to categorize patient comments against our list of five therapeutic affordances: self-presentation, connection, exploration, narration, and adaptation. These results will be the subject of a separate manuscript.

Furthermore, the posttest PROMs were analyzed using paired *t* tests and Fisher's exact test. In order to compute the PROMIS-PI data, raw scores were first translated into a T score for each patient. The T score rescales the raw scores into a calibrated and standardized score with a mean of 50 and standard deviation of 10. The value of hypothesis testing is limited due to the small sample size and given that potential covariates such as gender, age, and educational level were not able to be stratified [45].

## Results

### Study Design and Processes

#### Recruitment

During nine rounds of referral screening at RMH-PMS Direct Access Unit, 235 referrals were examined for suitability. A total of 138 out of 235 (58.7%) were deemed appropriate for possible study inclusion. A total of 55 out of 235 (23.4%) were inappropriate for inclusion based on priority status for medical intervention and/or medical status, planned discharge, psychological status, drug-seeking behavior, and/or cognitive impairment. A further 42 out of 235 (17.9%) were from non-English-speaking backgrounds (NESBs) and, thus, inappropriate for this particular study (see Table 2).

**Table 2:** Referral screening process for possible inclusion into the pilot study.

Round	Date (dd/mm/yy)	Assessed, n	Suitable, n	Inappropriate, n	Non-English speaking, n
1	15/04/14	58	41	11	6
2	13/05/14	32	18	5	9
3	03/06/14	12	6	1	5
4	10/06/14	31	12	16	3
5	24/06/14	32	22	5	5
6	10/07/14	8	6	0	2
7	29/07/14	16	11	2	3
8	19/08/14	25	11	8	6
9	02/09/14	20	10	7	3
Total, n (%)	N/A^a^	235 (100)	138 (58.7)	55 (23.4)	42 (17.9)

^a^N/A: not applicable

Multimedia Appendix 7 is the recruitment spreadsheet detailing success of the recruitment effort. Unreachable patients are shaded with grey (54/138, 39.1%); these represented roughly equal numbers of males (25/54, 46%) and females (29/54, 54%), while age range of nonresponders was relatively unremarkable. Contact was made with 84 out of 138 (60.9%) potentially suitable patients. Those indicated by the color red in Multimedia Appendix 7 were excluded (54/84, 64%). Reasons for exclusion are further broken down into *self* (19/54, 35%) or *external* (35/54, 65%). Table 3 shows reasons for exclusion. Major reasons for *self*-exclusion were as follows: no interest in participating and no connected devices. *External* reasons for exclusion included the following: patient moved to priority treatment, medically inappropriate, and non-English speaking.

**Table 3:** Self-exclusion and external reasons for nonparticipation.

Reason for nonparticipation		n (%)
**Self-exclusion (n=19)**		
	Not interested in participating	8 (42)
	No connected devices	4 (21)
	Prolonged computer use flares pain	3 (16)
	Time poor	2 (11)
	Against Internet and Facebook	1 (5)
	Confident with self-management	1 (5)
**External (n=35)**		
	Moved to priority treatment	12 (34)
	Medically inappropriate	8 (23)
	Non-English speaking	8 (23)
	Discharged from waiting list	5 (14)
	Duplicate referral	2 (6)

**Figure 1:**
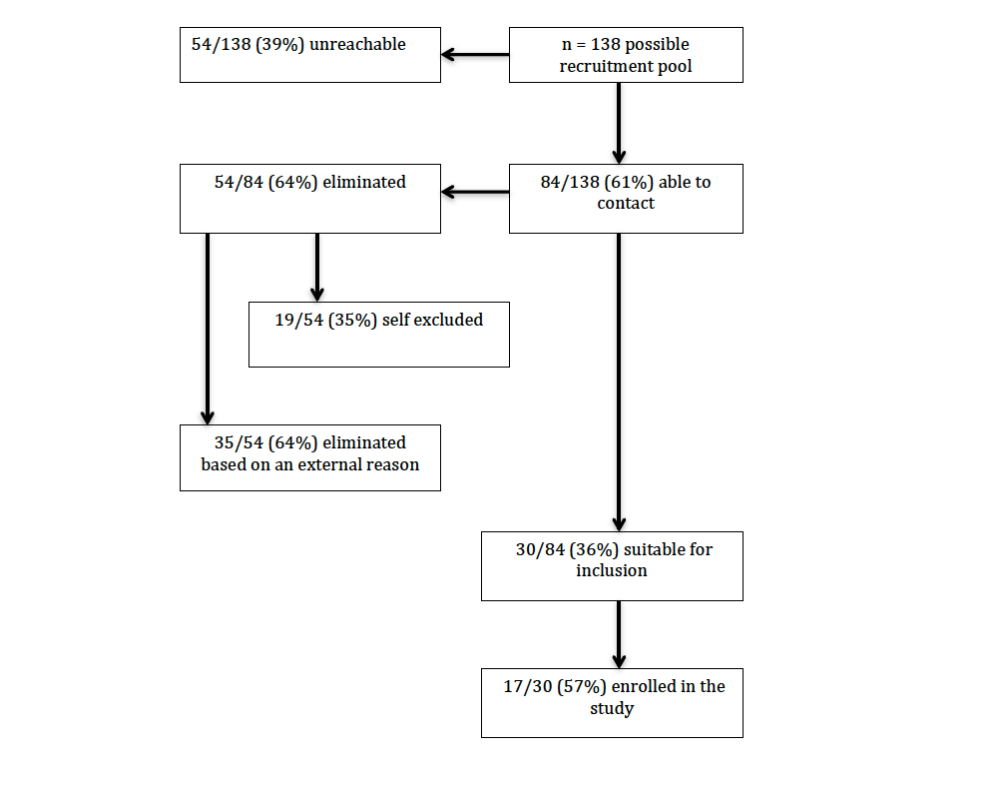
Recruitment process after screening referrals leading to final cohort.

This process left a total of 30 out of 84 (36%) patients fitting the inclusion criteria and interested in study participation. All 30 patients were emailed the study information for registration purposes; this process yielded a final study cohort of 17 out of 30—indicated in blue in Multimedia Appendix 7—who supplied pretest data, giving a participation rate of 57%. Those that did not register are highlighted in orange. The full recruitment process can be seen in Figure 1.

#### Participants

Demographics are presented in Table 4. The study included slightly more females (10/17, 59%). Age range was spread: 13 out of 17 (76%) patients were aged between 18 and 39 years, with only 1 patient aged older than 50 years. A total of 10 out of 17 (59%) patients reported never being married and 4 out of 17 (24%) were married/partnered. Education level also varied, with 9 out of 17 (53%) patients having completed high school or less and 8 out of 17 (47%) having obtained a university degree or higher. Work status indicated that more than half of the patients were not working due to ill health (9/17, 53%) and only 3 out of 17 (18%) patients were currently working full time.

Enrolled patients were asked to provide information about their chronic pain. A total of 9 out of 17 (53%) patients reported a duration of chronic pain between 1 and 5 years; 7 out of 17 (41%) reported pain duration of greater than 5 years. Low back pain was the primary ailment reported (8/17, 47%)—defined as upper/middle/lower back pain—whereas 3 out of 17 (18%) patients reported hip/leg/foot pain as their primary pains. Various offline treatment modalities—in the last 12 months—for pain management were noted. Doctor’s visits (16/17, 94%) and medication (15/17, 88%) were most reported. Physical therapies (ie, physiotherapy, massage, myotherapy) were next (13/17, 76%), followed equally by exercise classes and relaxation/meditation (8/17, 47%), and finally, psychology/counseling (5/17, 29%). Other free-text responses also highlighted acupuncture and walking.

Each patient was asked whether their pain was *flared* or *stable* at the time of the study. The majority (12/17, 71%) reporting *flared*, with 12 out of 17 (71%) patients reporting average day-to-day pain at a level of 6 to 7 out of 10 (mean 7.1, SD 1.2). Patients were also asked to indicate whether they had been formally diagnosed with a condition causing their pain and 10 out of 17 (59%) indicated this was the case. Fibromyalgia (3/17, 18%) and osteoarthritis (2/17, 12%) were most noted. Other recorded conditions included, but were not limited to, posttraumatic stress, temporomandibular joint syndrome, sciatica, and low back pain.

**Table 4:** Study demographics.

Patient characteristics (n=17)		n (%)
**Gender**		
	Male	7 (41)
	Female	10 (59)
**Age range in years**		
	18-29	7 (41)
	30-39	6 (35)
	40-49	3 (18)
	50-59	1 (6)
	60+	0 (0)
**Marital status**		
	Never married	10 (59)
	Currently married/partnered	4 (24)
	Separated/divorced/widowed	3 (18)
**Level of education**		
	High school or less	9 (53)
	College/university completed	6 (35)
	Postgraduate degree completed	2 (12)
**Work status**		
	Full time	3 (18)
	Part time	2 (12)
	Not working (ill health)	9 (53)
	Not working (other)	3 (18)

#### Engagement and Completion of the Study

Out of 17 patients, 16 were contactable, supplying data for this study (94% success rate).

A total of 12 patients out of 17 (71%) reported using the resources during the study. Based on the number of times each patient could be reached on the phone to collect data (n=38) and call durations of 15 minutes, on average, it is approximated that a rich dataset of 570 minutes or 9.5 hours of qualitative interview data was collected.

Study completion rate was calculated based on the number of completed posttest questionnaires received. Despite the success of the semistructured interviews, 9 out of 17 completed posttest questionnaires were received, giving a study completion rate of 53%. Out of 17 patients, 1 (6%) dropped out between study enrolment and first feedback phone call, 1 (6%) withdrew before final follow-up as she reported no longer needing pain management services, 2 (12%) submitted the final questionnaire but reported not having used the resources (hence, were eliminated from further analysis), and finally, 4 (24%) failed to submit the posttest questionnaire despite several attempts to contact them. No contact by patients was attempted during any phase of data collection requesting assistance completing the surveys. This satisfied the research team that the data collection tools were acceptable.

### Barriers to Engagement With the Social Media Resources

Coding of the semistructured interview data yielded eight separate engagement themes (see Table 5). Based on the number of times each barrier was identified, *time poor* and *low specificity of resources* to one’s condition were most noted. *Effects of medication* was also highlighted; patients noted that this was due to their sedative effect and/or impact on concentration. Only 2 out of 17 patients (12%) suggested access to the Internet was an issue.

Some of these barriers were also identified in the free-text responses of the posttest survey, for example, *low specificity of resources*. According to Patient SM021, “I have a more localized pain condition and most of the resources were for generalized conditions so I had trouble relating to many of them.” This was echoed by Patient SM112, who stated, “I think that because they weren’t about my pain and there were none about the pain I was going through I found it not very useful.”

**Table 5:** Barriers to engagement with the social media resources.

Theme	Participants (n=17), n (%)
Time poor	7 (41)
Low specificity of resources	6 (35)
Effects of medication	4 (24)
High pain levels	2 (12)
Pain-focused mentality	2 (12)
Internet access	2 (12)
Too much text-based information	1 (6)
Pain resolved	1 (6)

## Discussion

### Principal Findings

The aim of this study was to examine the pilot-testing of social media use with patients in the clinical setting, focusing on major aspects of the study design. The study also produced evidence to support social media use and PROs from earlier studies [26,27]. However, these findings are not the focus of this protocol paper.

### Study Design

The use of mixed methods in eHealth research is often advocated [46-48]. This study also presents a strong case for the complementarity of such a research design. The richness of both quantitative and qualitative datasets and their value to examining the effectiveness of pilot studies has been discussed within a similar research context to this study [32,36,49].

By combining empirical PROs data from PROMIS-PI, PSEQ, and from the social media-use instrument with the qualitative semistructured interview data and free-text responses about social media use from the posttest survey, a rich dataset was obtained. This becomes particularly useful with a small sample size, such as the one in this study. It helps to cross-validate findings and strengthen converging inferences [46].

Collecting empirical data about social media use was able to corroborate semistructured interview data. Similarly, data collected regarding patient perceptions toward each of the five therapeutic affordances of social media helped validate findings from a global online survey of PWCP [26,27]. This enhances the scope to generalize study findings in future research and across different study areas and conditions within similar domains.

### Recruitment

The recruitment procedure used in this study was extensive and demonstrates a targeted method aimed at recruiting the most suitable clinical cohort that might benefit from the intervention. It is the belief of the research team that the method for recruiting such a targeted cohort in this study is appropriate and would be scalable in a larger-scale randomized controlled trial (RCT) study. This is because the aim was to refine the most suitable cohort required to test the effectiveness of social media to positively impact PROs. This was not a study of readiness to adopt social media. A similar targeted approach has been seen in other pilot studies within a similar study domain [32,36,50].

However, targeted recruitment does highlight pertinent ethical considerations, such as “representativeness and selection.” Internet access and English language problems were two considerations noted from the RMH-PMS population. Similarly to this study, most social media interventions target Internet-competent, social media-using participants and/or people thinking about engaging in health behavior change [23,51,52]. While some may argue that this may limit the generalizability of findings, it has also been argued that chronic pain patients almost always self-select their own management (ie, medication, physiotherapy, exercises, surgery, etc). Hence, a self-selecting study design is still ecologically valid [11].

Demographic characteristics predicting participation in social media or other Web-based interventions for chronic disease management are also interesting and relevant. This has traditionally been skewed towards well-educated females of a relatively high socioeconomic status. Marital status and age are more varied but studies are typically slanted toward those in relationships/married and aged 30 to 60 years [53-55]. Literature has also previously reported that people living with chronic illness are typically more representative of lower socioeconomic status and educational level [51,56,57]. What was found in the present study is that the clinical setting is representative of a greater demographic spread and it was this clinical PWCP population that was of interest for examination. Patients were an equal mix of males and females, education level was generally lower (in this study, 9/17 [53%] completed high school or less), more than half were unemployed due to ill health (9/17, 53%), and age range varied from approximately 18 to 59 years. Earlier studies, such as that of Berman et al [33], reported significant improvements in PROs for older chronic pain patients (55+ years). They also reported minimal issues for participants navigating the online resources, suggesting that older adults in the chronic pain setting may also derive benefit from online interventions. In this study 19 out of 138 (13.8%) potentially suitable study recruits were aged 55 years or over.

### Engagement and Completion of the Study

According to Sheaves et al [36], the study completion rate of 53% (9/17) is comparable with other Web-based health studies. Reasons for attrition according to interview data about *barriers to engagement*, may have been *low specificity of resources* to one’s condition and *lack of time* to use the resources.

The semistructured interviews conducted monthly by telephone were an important tool in understanding reasons for engagement or nonengagement above. In contrast to overall completion rate, results showed that 94% (16/17) of patients were able to be engaged by phone. This enhanced collaboration between patients and the research team and placed greater emphasis on patient preferences and perceptions about using social media for their self-management. In other studies, such as that of Sheaves et al [36], email was used to collect data. The authors reported that lack of response to feedback emails was high. Hence, conducting semistructured interviews by telephone rather than via email may be a more successful data collection and patient engagement method in social media studies.

As noted from patient feedback, low specificity of resources to one’s condition negatively influenced engagement. It has been reported that often those living with chronic conditions have multiple comorbid manifestations and, hence, Web-based resources can fail to cover enough of their individualized needs for them to be deemed of significant value [58]. As could be seen from the research design, general chronic pain management social media resources were suggested to patients, not those specific to any one chronic condition (eg, low back pain, rheumatoid arthritis). This was decided based on current evidence-based approaches to chronic pain management, focusing on holistic multi-faceted, versus disease-specific, management [10]. The conflict observed between a desire to deliver evidence-based practice (EBP) and patient preferences for their own management highlights the need for greater emphasis on shared decision-making models between patient and clinician to achieve success in larger-scale studies in the future [18].

Suggesting certain social media resources as a starting point was done to provide some element of quality control and uphold ethical standards of care. However, freedom was encouraged for patients to use the resources as they wished and explore different avenues using the social media resources. Unlike many earlier studies conducted in this space, the aim of the present study was to more closely replicate social media use in day-to-day life (ie, open, engaging, collaborative, participatory) rather than to create a specific online intervention that dictates exactly what patients engage with and how they engage with it.

Finally, facilitated engagement of social media interventions, or involvement of clinicians, has been reported as another positive way to improve participation and intervention adherence. In turn, this has a positive flow onto PROs [9,20,53-55]. In Hoch et al [32], the entire pilot intervention was guided by a clinical nurse. This led to a completion rate of 24 out of 28 (86%). However, unlike this study, that of Hoch et al [32] was a social media intervention that translated a traditional face-to-face intervention into one delivered in a virtual world. The nurse had an integral role in delivering each session, thus mimicking offline management. Autonomous self-management was the goal for this study, not translating an intervention into an online one. Hence, using social media resources cannot be truly facilitated in the same manner. Empowering patients to make their own decisions about using social media in this manner also has positive connotations for study logistics, suggesting that fewer clinical staff would be required to run future larger-scale studies.

### Strengths and Limitations

#### Strengths

The foremost strength of this pilot study lay in the intervention design. The choice was to encourage participating patients to be more autonomous and decide on which social media to use and how to use them as part of self-management. This breaks away from conventional Web-based intervention design based on constrained, predefined online interventions. As discussed, this idea fits within a participatory model, suggesting, not prescribing, a Web-based intervention. It places greater weight on patients’ perceptions and preferences for health self-management online.

Web-based interventions in chronic pain traditionally collect data using several disparate PROMs. As seen and described in Buhrman et al [50], the decision to incorporate several legacy measures increases the risk of deriving findings due to chance. The authors also suggest that many chronic pain PROMs have a weak theoretical basis. Future research would benefit from utilizing standardized, validated, and generalizable measures to overcome such issues. This is another reason to advocate for the strength of using PROMs such as PROMIS within this research domain [39,40]. It is the belief of the research team that, to date, no similar studies have used PROMIS for outcome measurement in this context. PROMIS has been validated against a variety of measures and across a range of conditions. Hence, using this study as a benchmark, researchers interested in studying social media for chronic pain management will be able to compare findings across a range of contexts and conditions in the future.

#### Limitations

RCT designs are synonymous with robust health research. This method is strongly advocated in trials of effectiveness and has been employed in other Web-based chronic pain studies [33,50]. However, the decision was made to run this study as a single-arm trial with only an intervention group as numbers would not be sufficient to warrant an RCT at this stage. Similar designs and sample sizes have been seen in other social-media-in-chronic-disease pilot studies [32]. The recruitment process showed how difficult it is to recruit large numbers of patients into studies where no incentives are offered. As was seen, it was not until members of the clinical team began conducting recruitment phone calls that a rise in study interest was seen and, hence, enrolments. Future research warrants larger-scale studies to recruit sufficient numbers to use RCT designs to accurately test the effectiveness of social media use in the clinical management of chronic pain.

The decision to merely suggest social media resources, but allow patients to make their own decisions about which to use, meant that the resources patients actually used were not able to be verified. PROs collected may be in reference to a variety of social media resources, both reputable and not. Thus, study findings are open to interpretation bias. This is one of the reasons that emphasis is not placed on posttest PROMIS-PI and PSEQ findings in this study.

There is a trade-off between targeted recruitment of competent and enthusiastic social media-using chronic pain population members and ensuring all chronic pain patients who may benefit from social media can participate. Until social media interventions can better address the needs of chronic pain patients who suffer from a lack of Internet access, poor literacy skills, poor Internet literacy, and language barriers, they will always be biased toward self-selecting populations. Hence, any conclusions drawn from this pilot study regarding recruitment, intervention design, and engagement are in reference to the current sample only.

Other pilot studies [32,33] have reported completion rates slightly better than those of this study (9/17, 53%). However, both of these studies utilized incentives ranging from monetary amounts through to allowing participants to keep the supplied technology. This study did not offer incentives as a way to minimize selection bias, but their value is not discounted for future study as another way to enhance recruitment and/or decrease attrition.

### Recommendations and Conclusions

This pilot study has outlined key considerations for conducting social media interventional research in the clinical setting, in particular, study design, recruitment, and engagement.

Targeted recruitment of social media users indicates that enthusiastic, competent social media users may be still largely underrepresented on pain management services’ waiting lists. Therefore, these interventions may not yet be appropriate for all PWCP. Further work is required to ensure that those in need of online support will also be catered to by social media’s integration into clinical service models. In line with recommendations by Sheaves et al [36], future studies would be well advised to (1) include general practice sites in research, where patients may have more need for information (given that they are more likely to be earlier in the course of managing their pain) and (2) include eHealth literacy education, training, and support as part of care models for those who have low computer literacy skills, but may stand to benefit from online resources [35].

As patients become increasingly connected and active shared decision makers in their self-management, researchers would be advised to pay close attention to study designs that give patients greater flexibility and empower them to make decisions about the online resources they use. This is where the potential for social media sits above traditional Web-based interventions. Social media resources must actively engage patients as seen in this study. Finally, acknowledging patient preferences for resources that adequately address disease-specific needs is also a consideration.

Future research into the effectiveness and potential for social media use in the clinical management of chronic pain is warranted. While this study cannot ascertain what effect the use of social media resources can ultimately have on hospital waiting lists, a considered approach to conducting this type of research has been offered. Future studies need to focus on larger-scale, multicenter RCTs and involve patients in the intervention design in order to achieve desired effect sizes and for evidence to be sound.

## Appendix

Multimedia Appendix 1 Telephone transcript.

Multimedia Appendix 2 Patient educational material.

Multimedia Appendix 3 Selected study resources.

Multimedia Appendix 4 Pretest questionnaire.

Multimedia Appendix 5 Posttest questionnaire.

Multimedia Appendix 6 Semistructured interview template.

Multimedia Appendix 7 Recruitment list.

## Abbreviations

CBT: cognitive behavioral therapy
EBP: evidence-based practice
HRQOL: health-related quality of life
MNSI: Melbourne Networked Society Institute
N/A: not applicable
NESB: non-English-speaking background
PI: pain interference
PRO: patient-reported outcome
PROM: patient-reported outcome measure
PROMIS: Patient-Reported Outcome Measurement Information System
PROMIS-PI: Patient-Reported Outcome Measurement Information System—Pain Interference
PSE: pain self-efficacy
PSEQ: Pain Self-Efficacy Questionnaire
PWCP: people with chronic pain
QOL: quality of life
RCT: randomized controlled trial
RMH: Royal Melbourne Hospital
RMH-PMS: Royal Melbourne Hospital—Pain Management Services
