# Supplementary material for: Using Social Media While Waiting in Pain: A Clinical 12-Week Longitudinal Pilot Study
Source: JMIR Res Protoc. 2015 Aug 7;4(3):e101. doi: 10.2196/resprot.4621 (PMC4705018; doi:10.2196/resprot.4621)
Supplement: Multimedia Appendix 1 [file resprot_v4i3e101_app1.pdf]

Appendix 1: Telephone Transcript

“Social media use by chronic pain patients”

This document provides an outline of the content of proposed phone conversations inviting patients to participate in our study. Whilst we endeavour to provide a robust and accurate representation of the nature of the recruitment phone call, certain areas need to be discussed. These are outlined as follows:

- Whilst clinical staff at the Royal Melbourne Hospital – Royal Park Campus will attempt to steer the conversation in a consistent direction, it is anticipated that no two phone conversation will be identical
- Certain details discussed over the phone (such as one’s medical condition) may be private in nature, for this reason these details will be confidential between participant and clinician at the hospital and it is therefore ethically inappropriate to attempt to replicate this in a transcript.

Transcript:

Part 1): Direct Access Unit (care coordinators) call participants shortlisted as potentially suitable based on information contained within the referral, who have been referred to the chronic pain clinic to register the client.

At the same time the care coordinators explain to them the standard wait list times and ask them if they would be interested in taking part in a research project that might be of interest to people on the wait list. They ask if they would be happy for a research person the contact them with details.

Part 2): Call from investigators

Researcher: “Hello [name], it’s [name] from the chronic pain clinic at the Royal Melbourne Hospital. How are you today?”

“The reason I’m calling is because you recently spoke with my colleague on the care team and he/she mentioned a project that might be interest to people such as yourself, who are on the clinic’s wait list. I’m calling to discuss that we are trialling an Internet project”.

“Is this an ok time to talk?”

“I shouldn’t take up more than a few minutes of your time?

- If no, “is there a more suitable time to call?” [……………….]

“Please be assured that this call is confidential”

“We realise that this wait list is long and it’s a long time to be waiting in pain. That’s why we’re undertaking this research project”

“It’s a project together with health technology researchers from the medical school at Melbourne University”.

“Is it something you might be interested in having access to?”

yes / no

If yes, “Ok great. Are you ok with me asking you a few more questions about your Internet use?”

- Do you have regular access to the Internet?

yes / no

- Do you have a Facebook and/or Gmail account? You will need these to use the material we provide. If you don’t, are you at least willing to register accounts? and be bound to the Sites’ terms and conditions

yes / no

- Are you currently undertaking any Internet-based intervention to manage your pain? (if yes, get details but may result in exclusion)

yes / no

- Are you currently using online chronic pain resources on the Internet for your management of this condition? i.e. Facebook, blogs, etc (if yes, exclude – creates bias)

yes / no

……NO at any point = exclude

<u>**Competence questions**</u>

- Have you used the Internet (such as Google) to find information?

yes / no

- Have you sent emails including attachments before?

yes / no

- Have you ever posted messages to chat rooms, forums, or groups?

yes / no

- Have you ever used it to make phone calls? i.e. Skype

yes / no

- Ever shared files with friends of family?

yes / no

- Ever created a website or personal page?

yes / no

- Have you ever uploaded anything to a website (i.e. text, photos, music) such as Facebook, Twitter, YouTube, etc?

yes / no

- Changed security settings or preferences on your Internet browser?

yes / no

Score total out of 8 (Need at least a yes to 3-5) to continue.

[score = ……………..]

- ***If <3-5 need to explain the study is quite technical and they are not suitable for this research. Mention can provide them access to a website we recommend called "painhealth" from Western Australia Health that they can play with to ease into use of online pain resources + use while waiting for upcoming pain clinic aapointment.***

- ***If yes (3-5+): Suitable competence***

If participant is suitable………

"The research involves offering access to online pain resources for 12 weeks to improve your understanding of your pain and provide you with some help with your management while you wait to receive a place in the program."

Result: "I think you'll be well suited to manage this study."

"I need to pass you on some information about registering for the study"

"I'll need your email address so I can send you the link to the study information website and questionnaire. Do you have an email address?"

yes / no [……………………………………………………………………………..]

"The website goes into more detail and will give you more time to digest the project. If you have any questions after reading it, feel free to give me a call"

"If you are interested and happy to participate, you'll need to fill out the Before-Study Questionnaire to enter yourself into the study."

"The people who register will be randomly allocated to one of two groups. There is a difference between the 2 groups. The first group will be using the online resources straight away for 12 weeks. The second group will also be given access to the resources to use freely but this will occur after the 12 weeks. This group will continue management as usual during the 12 week period."

"For those assigned to the resources/first group, we'll be scheduling some short information sessions where we'll introduce material and explain the study in more detail"

"As a final note, all information provided and sourced from you will be strictly confidential and in no way affect your treatment at the hospital or entitlement to care in the pain clinic. This is true whether you participate or not."

"Do you have any questions at this point?"

"Please check your email over the next day or so for an email from us. It will contain the link to the study information to go over, consent form and Before-Study Questionnaire. "

"Have a final read of everything and if you are happy please fill in the questionnaire at the end of the information to confirm your enrolment in the study"

"We'll then get in touch with further information"

"Thanks for your time. Have a nice day"

END
