# Supplementary material for: Using Social Media While Waiting in Pain: A Clinical 12-Week Longitudinal Pilot Study
Source: JMIR Res Protoc. 2015 Aug 7;4(3):e101. doi: 10.2196/resprot.4621 (PMC4705018; doi:10.2196/resprot.4621)
Supplement: Multimedia Appendix 2 [file resprot_v4i3e101_app2.pdf]

Appendix 2: Patient Educational Material

Thank you for participating in this project.

The purpose of this information is to introduce you to the resources you will have access to.

The study resources can be found here:

RESOURCE	LINK	NOTES
**Facebook**	https://www.facebook.com/SurvivingChronicPain	This is a Facebook chronic pain page with over 27,000 members
**YouTube**	http://www.youtube.com/user/painHEALTH/videos	These are a selection of videos by people with chronic and videos from health professionals talking about pain management
**Blogs:**	http://tinyurl.com/q2ndryg	Blogger platform to create your own blog
**i) Living With Chronic Pain**	http://blogs.psychcentral.com/chronic-pain/	
**ii) Chronic Pain Blog**	http://www.everydayhealth.com/columns/life-with-chronic-pain/	
**iv) Seeking Equilibrium**	http://rosemaryl.blogspot.com.au/	

Further information

Here are links to short videos presented by Mark Merolli that might be of some help if you require assistance registering for Gmail, Facebook, YouTube and Blogger.

Gmail: http://www.youtube.com/watch?v=UGbw1J3yZx8

YouTube: http://www.youtube.com/watch?v=lKBsELp9kDg

Facebook: http://www.youtube.com/watch?v=zDHscZDhZ-8

Blogs: http://www.youtube.com/watch?v=8KLlxbsXiX4

*Please note, you will be contacted at monthly intervals by a member of the research team to monitor progress and for you to ask any technical questions related to the study (please note, these phone calls will not be consulatory in nature).

Please see below list of contacts:

*Please ask questions if there's anything you don't understand.

Ms. Marama Dunne
Clinical Nurse Consultant
The Royal Melbourne Hospital - Royal Park Campus
Phone: 0407 141 563

Mr. Steve Mantopoulos
Allied Health Pain Clinic Co-ordinator
The Royal Melbourne Hospital - Royal Park Campus
Phone: 8387 2194

Mr. Mark Merolli
Health & Biomedical Informatics Centre
The University of Melbourne
Phone: 8387 2194

Dr. Malcolm Hogg
Head of Pain Services
Royal Melbourne Hospital – Royal Park Campus
Phone: 8387 2194

Disclaimer:

Be aware that acting upon information you read from the Internet may make your condition worse and acting upon this information could possibly lead to inappropriate decisions being made about your management and may be harmful. Internet resources and the resources used in this study are to be used as guides only and are not a substitute for medical care and advice. Please contact your doctor for further advice.
