# Supplementary material for: Using Social Media While Waiting in Pain: A Clinical 12-Week Longitudinal Pilot Study
Source: JMIR Res Protoc. 2015 Aug 7;4(3):e101. doi: 10.2196/resprot.4621 (PMC4705018; doi:10.2196/resprot.4621)
Supplement: Multimedia Appendix 3 [file resprot_v4i3e101_app3.pdf]

Appendix 3: Selected Study Resources

a) YouTube – Chronic Pain Story Videos

Use of YouTube was to suggest watching pain management videos but also to give patients the opportunity to film and upload videos. The videos used in this study were from the '**Pain Health' YouTube channel.** These videos have been produced by the Western Australian Department of Health by Health Professionals.

http://www.youtube.com/user/painHEALTH/videos

Criteria for choosing these videos rather than the full website was that the website represents a web 1.0 model of web-based resources. It is a stand-alone resource for information provision, not interaction and collaboration. The videos found on the website on the other hand were social, hosted on YouTube. They are videos from both patients and health professionals discussing management of chronic pain and personal experiences of living with chronic pain.

Patients were encouraged to sign in to YouTube to give them the opportunity to post their own pain story videos if they wished (rather than solely watching the videos), share, rate and comment.

Investigating use of the YouTube videos allows us to further investigate therapeutic affordances such as:

- Exploration: how well the videos guide the participant to useful information and whether the information provided by the videos of health professionals are of value
- Connection: value of having the videos available wherever and whenever one chooses
- Narration: most videos are very much geared to the narrative experience as patients detail their stories of chronic pain
- Adaptation: we can examine for what reason participants used the videos to source information on chronic pain and how often this occurred

b) Facebook - Surviving Chronic Pain Page

https://www.facebook.com/SurvivingChronicPain

Decision criteria to include "Surviving Chronic Pain on Facebook" was based on several factors.

- World's largest moderated chronic pain support group
- The page has over 30,000 likes and over 5.5 thousand people actively talking about it
- Originated from the USA but has global members, including Australia
- It receives regular activity (i.e. on an hourly basis)

- It was started by people with chronic pain (PWCP) for PWCP so it is active and not commercially focused
- It is peer to peer driven/facilitated
- Posts and questions receive timely answers
- It is moderated and any member's questions go through a facilitator or moderator before being posted
- It links to the a 'Surviving Chronic Pain' Facebook Group, which is a closed group that provides even deeper discussion and contact with others also living with chronic pain
- It contains other social features such as a chat room to communicate online with other members

d) Blogs - Selection of Chronic Pain Blogs

To be consistent and rigorous when including blogging resources and also to best replicate how most people would search and find blogs on the Internet, we searched "Google" to identify the best chronic pain blogs. Search was performed using "chronic pain blogs", "top chronic pain blogs", "best chronic pain blogs". No specific hub or reference for chronic pain blogs was found. However, using this method, we were able to generate a list of chronic pain blogs to shortlist. To decide which blogs would be suggested for use, we elected to use the following inclusion criteria: blog found on the first page of search results, blog found across all search strings used, active posting in the last 12 months. The following blogs were suggested:

- Living With Chronic Pain Blog (http://blogs.psychcentral.com/chronic-pain/)
- Chronic Pain Blog (http://www.everydayhealth.com/columns/life-with-chronic-pain/)
- Seeking Equilibrium (http://rosemaryl.blogspot.com.au/)

Of these three blogs, one was written and monitored by a social worker, another was written and monitored by a nurse and the third was written by a chronic pain patient and had received multiple blogging awards.

In a similar way to YouTube, blogging allows for active patients to create and maintain their own blog. We encouraged patients to create, maintain and share their own chronic pain blog if they wished. Patients were introduced to "Blogspot', a simple, user friendly blogging platform powered by Google and accessible using a Gmail account.
