# Supplementary material for: Using Social Media While Waiting in Pain: A Clinical 12-Week Longitudinal Pilot Study
Source: JMIR Res Protoc. 2015 Aug 7;4(3):e101. doi: 10.2196/resprot.4621 (PMC4705018; doi:10.2196/resprot.4621)
Supplement: Multimedia Appendix 4 [file resprot_v4i3e101_app4.pdf]

Social Media Use in Chronic Pain Management - Before Study

PATIENT INFORMATION

Ethics HREC ID 2014.043

Priniciple Researchers: Dr. Malcolm Hogg, Dr. Charles Kim, Dr. Kathleen Gray, Mr. Mark Merolli, Prof Fernando Martin-Sanchez

Thank you for your interest in participating in our research project. You have been approached to participate in this study because you are currently on the wait-list to access the outpatient pain clinic at the Royal Melbourne Hospital - Royal Park Campus.

The aim of this study is to better understand how you use different **Internet resources in relation to your pain**. It will assess which resources you use, how you use them and what you believe is most useful. We will also examine pain outcomes. Please note that your involvement in this project will not affect your position in the wait-list or affect your waiting time to access a place in the pain program.

The Hospital and University's Human Research Ethics Committees have approved this project.

Please read this information carefully. It tells you about the research project and explains what is involved. Understanding what is involved will help you decide if you want to take part. Ask questions about anything you don't understand or want to know more about.

Participation in this research is voluntary. If you don't wish to take part, you don't have to. You will receive the best possible care whether you take part or not.

If you do decide you are happy to take part, the final page of this document is the consent form. By clicking next from the consent form to the Before-Study Questionnaire and submitting it, you are indicating that you:

• Understand what you have read;
• Consent to take part in the research project;
• Consent to participate in the research processes that are described;
• Consent to the use of your personal and health information as described

*You can print this Participant Information and Consent Form to keep.

What is the purpose of this research project?

**You will have discussed your suitability for the study in your phone call from clinical investigators at the hospital. From this phone call, you confirmed that:*

• You can speak, read and write English well
• You have regular Internet access and are able to use the Internet reasonably well
• You are willing to register with Gmail and Facebook (if you don't already have accounts) and be bound to the Sites' terms and conditions
• You are not currently participating in any online intervention to manage your pain
• You are not currently using online resources for chronic pain management

We will collect information from you, including your answers to a range of questions, to better

understand your use of Internet resources to manage your pain, and its impact on your condition. These answers may be used to guide treatment in the future and to measure any benefits of using Internet resources as part of pain management. We realize that it is hard to properly measure the effect of using the Internet on your pain. However, the project aims to assess suitability of providing supportive resources to people such as you as you wait to access the chronic pain clinic.

This study is being conducted as part of a PhD research project from the Health & Biomedical Informatics Centre (Melbourne Medical School, the University of Melbourne) in conjunction with us at the hospital.

......please continue over page

PATIENT INFORMATION

What does participation in this research project involve?

The study runs for **12 weeks**. If you agree to participate, **please click 'next' to view the consent form**. By clicking through from the consent form you will be guided to the **Before-Study Questionnaire** where you will be asked questions regarding basic information about yourself and your pain. We anticipate our questionnaires to take about 15-20 minutes to complete. Completion of this questionnaire implies your consent to be involved in the study.

Collecting your answers to these questions aims to help us better understand your pain, its impact and how well suited different Internet resources are to improving your pain. These answers will provide insight into the benefits of adding Internet resources to pain management. We realize that it is hard to properly measure the effect of using the Internet on your pain. However, it will give us a better understanding of the ways people with chronic pain use the Internet and how it impacts pain.

After submitting your Before-Study Questionnaire, we will contact you again by phone to finalise details.

You will be:

• Completing the study over the 12 week period
• Provided with a handful of pre-selected online resources (selected and approved by the research team) (including YouTube videos, a Facebook support page and a selection of pain blogs) for you to visit and use over the 12 week period
• Provided with links to the resources and brief instructional videos to use later as you need
• You will be contacted at monthly intervals by clinic staff at the hospital to touch base about your progress (please note: these will not be pain consultation sessions)
• When you complete the study, you will be required to complete the Follow-up Questionnaire to assess your pain and use of the resources provided
o *We will also invite you to save and email us screen shots that show different areas of your usage (any identifying information will be removed)*

*<u>*Please note:</u> You are free to terminate accounts on Gmail and Facebook at any time*

You will not be paid for your participation in this research

......please continue over the page

PATIENT INFORMATION

What are the possible benefits?

We can't guarantee or promise any benefits from participation in this research. However, participating in the study may be beneficial towards improving your understanding about pain in order to manage it more effectively. Also, your participation may assist us in providing improved pain services in the future.

What are the possible risks?

Be aware that resources, such as those used in this project can sometimes contain incorrect information, and in the worst case scenario this can lead to inappropriate decisions being made about your pain management. Internet resources and the resources used in this study are to be used as guides only and are not a substitute for medical care and advice If you are unsure about any particular information you found online during this project, please consult your doctor.

You should be aware that responding to questionnaires about your pain or feelings regarding its effects may cause some people distress.

If you become upset or distressed as a result of participation in the research, the researchers are able to arrange for counselling or other appropriate support. Any counselling or support will be provided by staff that are not members of the research team. In addition, you may prefer to suspend or end your participation in the research if distress occurs.

Are there alternatives to participation?

Participation in this research is not compulsory. You may begin completing the questionnaire but chose not to submit. You may choose not to complete the questionnaire, which will not stop you being offered care through our service.

Do I have to take part in this research project?

Participation in any research project is voluntary. If you do not wish to take part you don't have to. If you decide to take part and later change your mind, you are free to withdraw from the project at any stage. However, please note that to withdraw after submitting the Follow-up Questionnaire, you would need to contact the researchers.

Your decision whether to take part or not to take part, or to take part and then withdraw, will not affect your management at the Royal Melbourne Hospital Chronic Pain Clinic or your relationship with the hospital.

What if I withdraw from this research project?

If you do withdraw, please notify Mr. Steven Mantopoulos to let him know (contact information in section 13 of this document).

Any information that you have already submitted will be kept by the researchers. This is to make sure that the results of the research can be measured properly. If you don't want this to occur, you should choose to not participate.

Could this research project be stopped unexpectedly?

We don’t expect this project to stop unexpectedly. A notice to complete the Follow-up Questionnaire will occur 12 weeks after you begin (that is after you submit the Before-Study Questionnaire)

How will I be informed of the results of this research project?

The results of this research project may be used to plan improvements to pain services in the future and will possibly be published in medical journals and presented at conferences. Whilst we do not plan to notify all participants with a summary of the results, we would be happy to provide the results upon written request.

......please continue over the page

PATIENT INFORMATION

What will happen to information about me?

Any information collected for the purpose of this research project that can identify you will be treated as confidential and securely stored. It will be disclosed only with your permission, or as permitted by law. Information will be stored in paper form in a locked filing cabinet, and computer form in a password-protected network, at the Health and Biomedical Informatics Centre, the University of Melbourne for 7 years and be accessible only to investigators participating in the project. We will require this stored information to include your name and other identifying details (eg. hospital record number, email address) in order to track your progress through the study, which is part of usual practice. This information will be used to remind us to prompt you to complete the Follow-up Questionnaire. Your information will be coded and all identifying information will be removed at the end of the study, before being stored as above. The code linking your details to any of this stored information will be stored at the Health and Biomedical Informatics Centre.

In any publication and/or presentation, information will be provided in a way that you can’t be identified, except with your permission.

Information about your participation in this research project may be recorded in your health records, but your decision to participate in the study or not will not affect your access to services at the hospital.

How can I access my information?

In accordance with relevant Australian and/or Victorian privacy and other relevant laws, you have the right to access the information collected and stored by the researchers about you. You also have the right to request that any information, with which you disagree, be corrected. Please contact one of the researchers named at the end of this document if you would like to discuss access to your information.

Is this research project approved?

The ethical aspects of this research project have been approved by the Human Research Ethics Committees of Melbourne Health (Royal Melbourne Hospital) and the University of Melbourne.

This project will be carried out according to the National Statement on Ethical Conduct in Human Research (2007) produced by the National Health and Medical Research Council of Australia. This statement has been developed to protect the interests of people who agree to participate in human research studies.

Who can I contact?

Who you may need to contact will depend on the nature of your query, therefore, please note the following:

<u>For further information:</u>

If you want any further information concerning this project or if you have any medical problems

which may be related to your involvement (for example, any side effects), you can contact the researchers below:

***Please note: you should print this list before continuing (alternatively, you will receive a copy by email)**

Ms. Marama Dunne
Clinical Nurse Consultant
The Royal Melbourne Hospital - Royal Park Campus
Phone: 0407 141 563

Mr. Steve Mantopoulos
Allied Health Pain Clinic Co-ordinator
The Royal Melbourne Hospital - Royal Park Campus
Phone: 8387 2194

Mr. Mark Merolli
Health & Biomedical Informatics Centre
The University of Melbourne
Phone: 8387 2194

Dr. Malcolm Hogg
Head of Pain Services
Royal Melbourne Hospital – Royal Park Campus
Phone: 8387 2194

For complaints:

If you have any complaints about any aspect of the project, the way it is being conducted or any questions about being a research participant in general, then you may contact:
Name: Jessica Turner, Manager Melbourne Health Human Research Ethics Committee, Telephone Number 9342 8530

......continue to consent form

CONSENT

I have read, or have had read to me in a language that I understand, this document and I understand the purposes, procedures and risks of this research project as described within it.

I have had an opportunity to ask questions and I am satisfied with the answers I have received.

I freely agree to participate in this research project as described.

I understand that I can print a copy of this document to keep.

***By clicking 'next' you will be taken to the Before-Study Questionnaire. By submitting answers to this questionnaire you are indicating that you have read and understood this information and the information contained in this document, and thus agree to participate in this study.**

Before Study - **DEMOGRAPHIC INFORMATION (information about you)**

Please read the instructions carefully before answering the questions.

Thank you for your time

***1. What is your study enrollment number? (this number must be in the format SMxx)**

***2. What is your gender?**

- ◯ Female
- ◯ Male

***3. Please tell us your age range (choose what is most appropriate):**

- ◯ 18-29
- ◯ 30-39
- ◯ 40-49
- ◯ 50-59
- ◯ 60+

***4. What is your current marital status?**

- ◯ Never married
- ◯ Married/Partnered
- ◯ Separated
- ◯ Divorced
- ◯ Widowed

***5. What is the highest level of education that you have completed?**

- ◯ High school or less
- ◯ College/university completed
- ◯ Post-graduate degree completed

***6. What is your current work status?**

- ◯ Full-time
- ◯ Part-time
- ◯ Not working due to ill health
- ◯ Not working for other reasons

Before Study - **INFORMATION ABOUT YOUR CONDITION**

We would like to ask you some questions about your pain and/or other medical conditions

***7. How long have you had your pain?**

- 3 - 6 months
- 6 months - 1 year
- 1 - 5 years
- 5 - 10 years
- 10 years +

***8. What is the main location of your pain? (if you have more than one, please indicate the one that is currently most troublesome)**

	Pain locations:
Please select:	

Other (please specify)

***9. Please select the treatments you have been having/using for your pain in the last year (you can select more than one):**

- Medication
- Doctor visits
- Physical Therapy (i.e. physiotherapy, osteopathy, myotherapy, etc)
- Exercise classes
- Psychology/counseling
- Relaxation/meditation

Other (please specify)

***10. In regards to your average day-to-day level of pain, would you say at the moment your pain is:**

- Flared-up
- Stable

***11. Have you have been formally diagnosed with a condition that has led to your pain?**

◯ Yes

◯ No

◯ Don't know

*** 12. If you selected 'yes' to the previous question, please select the condition from the drop-down menu**

	Medical conditions:
Please select:	

Other (please specify)

Before Study - **PAIN INTERFERENCE**

The following questions relate specifically to your pain and how it **INTERFERES** with living your life

Please respond to each item by marking one box per row. Follow the prompts

IN THE PAST SEVEN DAYS....

13. How would you rate your pain on average?

	0 (no pain)	1	2	3	4	5	6	7	8	9	10 (worst imaginable pain)
Please select:	◯	◯	◯	◯	◯	◯	◯	◯	◯	◯	◯

*** 14. How much did pain interfere with your enjoyment of life?**

	Not at all	A little bit	Somewhat	Quite a bit	Very much
Please select:	◯	◯	◯	◯	◯

*** 15. How much did pain interfere with your ability to participate in social activities?**

	Not at all	A little bit	Somewhat	Quite a bit	Very much
Please select:	◯	◯	◯	◯	◯

*** 16. How much did pain interfere with your relationships with other people?**

	Not at all	A little bit	Somewhat	Quite a bit	Very much
Please select:	◯	◯	◯	◯	◯

*** 17. How much did pain interfere with your family life?**

	Not at all	A little bit	Somewhat	Quite a bit	Very much
Please select:	◯	◯	◯	◯	◯

*** 18. How much did pain feel like a burden to you?**

	Not at all	A little bit	Somewhat	Quite a bit	Very much
Please select:	◯	◯	◯	◯	◯

*** 19. How often did pain make you feel anxious?**

	Never	Rarely	Sometimes	Often	Always
Please select:	◯	◯	◯	◯	◯

*** 20. How often did pain make you feel depressed?**

	Never	Rarely	Sometimes	Often	Always
Please select:	◯	◯	◯	◯	◯

*** 21. How much did pain interfere with your day to day activities?**

	Not at all	A little bit	Somewhat	Quite a bit	Very much
Please select:	◯	◯	◯	◯	◯

***22. How much did pain interfere with your household chores?**

	Not at all	A little bit	Somewhat	Quite a bit	Very much
Please select:	◯	◯	◯	◯	◯

***23. How much did pain interfere with your ability to work (include work at home)?**

	Not at all	A little bit	Somewhat	Quite a bit	Very much
Please select:	◯	◯	◯	◯	◯

***24. How difficult was it for you to take in new information because of pain?**

	Not at all	A little bit	Somewhat	Quite a bit	Very much
Please select:	◯	◯	◯	◯	◯

***25. How much did pain interfere with your ability to concentrate?**

	Not at all	A little bit	Somewhat	Quite a bit	Very much
Please select:	◯	◯	◯	◯	◯

***26. How much did pain make it difficult to fall asleep?**

	Not at all	A little bit	Somewhat	Quite a bit	Very much
Please select:	◯	◯	◯	◯	◯

***27. How often did pain prevent you from sitting for more than 30 minutes?**

	Never	Rarely	Sometimes	Often	Always
Please select:	◯	◯	◯	◯	◯

***28. How often did pain prevent you from standing for more than 30 minutes?**

	Never	Rarely	Sometimes	Often	Always
Please select:	◯	◯	◯	◯	◯

***29. How often did pain prevent you from walking more than 1 mile (1.6 km)?**

	Never	Rarely	Sometimes	Often	Always
Please select:	◯	◯	◯	◯	◯

Before Study - **CONFIDENCE TO SELF-MANAGE YOUR PAIN**

The following section relates specifically to your **CONFIDENCE** with self-management despite your pain:

For each of the following statements please choose the number that corresponds to your confidence that you can do the tasks regularly at the present time

***please note:** this is a 1-6 scale, different to the previous section you completed

Please respond to each item by marking one box per row

We would like to know how confident you are in doing certain activities **AT PRESENT**

*** 30. I can enjoy things, despite the pain**

	0 (not confident at all)	1	2	3	4	5	6 (completely confident)
Please select:	◯	◯	◯	◯	◯	◯	◯

*** 31. I can do most of the household chores (e.g. tidying-up, washing dishes, etc), despite the pain**

	0 (not confident at all)	1	2	3	4	5	6 (completely confident)
Please select:	◯	◯	◯	◯	◯	◯	◯

*** 32. I can socialize with my friends or family members as often as I used to, despite the pain**

	0 (not confident at all)	1	2	3	4	5	6 (completely confident)
Please select:	◯	◯	◯	◯	◯	◯	◯

*** 33. I can cope with my pain in most situations**

	0 (not confident at all)	1	2	3	4	5	6 (completely confident)
Please select:	◯	◯	◯	◯	◯	◯	◯

***34. I can do some form of work, despite the pain ("work" includes housework, paid and unpaid work)**

	0 (not confident at all)	1	2	3	4	5	6 (completely confident)
Please select:	◯	◯	◯	◯	◯	◯	◯

***35. I can still do many of the things I enjoy doing, such as hobbies or leisure activity, despite the pain**

	0 (not confident at all)	1	2	3	4	5	6 (completely confident)
Please select:	◯	◯	◯	◯	◯	◯	◯

***36. I can cope with my pain without medication**

	0 (not confident at all)	1	2	3	4	5	6 (completely confident)
Please select:	◯	◯	◯	◯	◯	◯	◯

***37. I can still accomplish most of my goals in life, despite the pain**

	0 (not confident at all)	1	2	3	4	5	6 (completely confident)
Please select:	◯	◯	◯	◯	◯	◯	◯

***38. I can live a normal lifestyle, despite the pain**

	0 (not confident at all)	1	2	3	4	5	6 (completely confident)
Please select:	○	○	○	○	○	○	○

***39. I can gradually become more active, despite the pain**

	0 (not confident at all)	1	2	3	4	5	6 (completely confident)
Please select:	○	○	○	○	○	○	○

END OF BEFORE STUDY QUESTIONS

Thank you for your responses. You have completed the before study questionnaire. We appreciate you taking the time.

You will be contacted to complete the final questionnaire in 12 weeks
