# Supplementary material for: Using Social Media While Waiting in Pain: A Clinical 12-Week Longitudinal Pilot Study
Source: JMIR Res Protoc. 2015 Aug 7;4(3):e101. doi: 10.2196/resprot.4621 (PMC4705018; doi:10.2196/resprot.4621)
Supplement: Multimedia Appendix 5 [file resprot_v4i3e101_app5.pdf]

After Study Questionnaire - Social Media Use in Chronic Pain

PATIENT INFORMATION

Thank you for participating in this project with us. Your feedback has been very insightful.

We look forward to receiving these final responses and seeing you at the clinic.

Clicking "next" will take you to the questions. They ask about your current pain and also about the online resources used.

Priniciple Researchers: Dr. Malcolm Hogg, Dr. Charles Kim, Dr. Kathleen Gray, Mr. Mark Merolli, Prof Fernando Martin-Sanchez

Ethics HREC ID 2014.043

After Study Questionnaire - Social Media Use in Chronic Pain

Follow-up - **PAIN INTERFERENCE**

***These questions are a follow up to those you completed in the before-study questionnaire**

The following questions relate specifically to your pain and how it **INTERFERES** with living your life

Please respond to each item by marking one box per row. Follow the prompts

***1. What is your study enrollment number? (this number must be in the format SMxxx)**

IN THE PAST SEVEN DAYS....

***2. How would you rate your pain on average?**

	0 (no pain)	1	2	3	4	5	6	7	8	9	10 (worst imaginable pain)
Please select:											

***3. How much did pain interfere with your enjoyment of life?**

	Not at all	A little bit	Somewhat	Quite a bit	Very much
Please select:					

***4. How much did pain interfere with your ability to participate in social activities?**

	Not at all	A little bit	Somewhat	Quite a bit	Very much
Please select:					

***5. How much did pain interfere with your relationships with other people?**

	Not at all	A little bit	Somewhat	Quite a bit	Very much
Please select:					

***6. How much did pain interfere with your family life?**

	Not at all	A little bit	Somewhat	Quite a bit	Very much
Please select:					

***7. How much did pain feel like a burden to you?**

	Not at all	A little bit	Somewhat	Quite a bit	Very much
Please select:					

***8. How often did pain make you feel anxious?**

	Never	Rarely	Sometimes	Often	Always
Please select:					

***9. How often did pain make you feel depressed?**

	Never	Rarely	Sometimes	Often	Always
Please select:	○	○	○	○	○

***10. How much did pain interfere with your day to day activities?**

	Not at all	A little bit	Somewhat	Quite a bit	Very much
Please select:	○	○	○	○	○

***11. How much did pain interfere with your household chores?**

	Not at all	A little bit	Somewhat	Quite a bit	Very much
Please select:	○	○	○	○	○

***12. How much did pain interfere with your ability to work (include work at home)?**

	Not at all	A little bit	Somewhat	Quite a bit	Very much
Please select:	○	○	○	○	○

***13. How difficult was it for you to take in new information because of pain?**

	Not at all	A little bit	Somewhat	Quite a bit	Very much
Please select:	○	○	○	○	○

***14. How much did pain interfere with your ability to concentrate?**

	Not at all	A little bit	Somewhat	Quite a bit	Very much
Please select:	○	○	○	○	○

***15. How much did pain make it difficult to fall asleep?**

	Not at all	A little bit	Somewhat	Quite a bit	Very much
Please select:	○	○	○	○	○

***16. How often did pain prevent you from sitting for more than 30 minutes?**

	Never	Rarely	Sometimes	Often	Always
Please select:	○	○	○	○	○

***17. How often did pain prevent you from standing for more than 30 minutes?**

	Never	Rarely	Sometimes	Often	Always
Please select:	○	○	○	○	○

***18. How often did pain prevent you from walking more than 1 mile (1.6 km)?**

	Never	Rarely	Sometimes	Often	Always
Please select:	○	○	○	○	○

Follow-up - **CONFIDENCE TO SELF-MANAGE YOUR PAIN**

The following section relates specifically to your **CONFIDENCE** with self-management despite your pain:

For each of the following statements please choose the number that corresponds to your confidence that you can do the tasks regularly at the present time

***please note:** this is a 1-6 scale, different to the previous section you completed

Please respond to each item by marking one box per row

We would like to know how confident you are in doing certain activities **AT PRESENT**

***19. I can enjoy things, despite the pain**

	0 (not confident at all)	1	2	3	4	5	6 (completely confident)
Please select:	○	○	○	○	○	○	○

***20. I can do most of the household chores (e.g. tidying-up, washing dishes, etc), despite the pain**

	0 (not confident at all)	1	2	3	4	5	6 (completely confident)
Please select:	○	○	○	○	○	○	○

***21. I can socialize with my friends or family members as often as I used to, despite the pain**

	0 (not confident at all)	1	2	3	4	5	6 (completely confident)
Please select:	○	○	○	○	○	○	○

***22. I can cope with my pain in most situations**

	0 (not confident at all)	1	2	3	4	5	6 (completely confident)
Please select:	○	○	○	○	○	○	○

***23. I can do some form of work, despite the pain ("work" includes housework, paid and unpaid work)**

	0 (not confident at all)	1	2	3	4	5	6 (completely confident)
Please select:	○	○	○	○	○	○	○

***24. I can still do many of the things I enjoy doing, such as hobbies or leisure activity, despite the pain**

	0 (not confident at all)	1	2	3	4	5	6 (completely confident)
Please select:	○	○	○	○	○	○	○

***25. I can cope with my pain without medication**

	0 (not confident at all)	1	2	3	4	5	6 (completely confident)
Please select:	○	○	○	○	○	○	○

***26. I can still accomplish most of my goals in life, despite the pain**

	0 (not confident at all)	1	2	3	4	5	6 (completely confident)
Please select:	○	○	○	○	○	○	○

***27. I can live a normal lifestyle, despite the pain**

	0 (not confident at all)	1	2	3	4	5	6 (completely confident)
Please select:	○	○	○	○	○	○	○

***28. I can gradually become more active, despite the pain**

	0 (not confident at all)	1	2	3	4	5	6 (completely confident)
Please select:	○	○	○	○	○	○	○

Follow-up - **ONLINE RESOURCE USE**

In this section, please tell us about the **INTERNET RESOURCES** used in this study

Follow-up - **SURVIVING CHRONIC PAIN FACEBOOK PAGE**

The following questions relate specifically to the **SURVIVING CHRONIC PAIN Facebook Page**

29. Please comment if you particularly LIKED anything about using FACEBOOK as part of your pain management in this study (i.e. features, activities, information, uses, etc)

30. Please comment if you particularly DISLIKED anything about using FACEBOOK as part of your pain management in this study (i.e. features, activities, information, uses, etc)

***31. Did SURVIVING CHRONIC PAIN on Facebook lead you to any other online resources? (e.g. other Facebook pages, blogs, videos, wikis, websites)**

- Yes
- No

*** 32. If 'yes' to the previous question, please briefly comment on what other types of online resources you came across from using FACEBOOK**

*** 33. How often did you visit SURVIVING CHRONIC PAIN on Facebook during this study?**

- At least daily
- At least weekly
- At least monthly
- Less often
- Never

*** 34. What SURVIVING CHRONIC PAIN on Facebook activities did you do? (you can select more than one):**

- ☐ Post an update to share your story
- ☐ Ask a question
- ☐ Respond to someone's posting or question
- ☐ Read or browse people's postings and experiences
- ☐ Like a comment or post
- ☐ Private message someone
- ☐ Add a friend/connection from the page
- ☐ Share someone's posting with others
- ☐ Use one of the chat rooms
- ☐ None of the above

Other (please specify)

Thinking about the FACEBOOK activities that you did, please answer the following statements..

***35. I was conscious to control the amount and sorts of things that other people knew about me**

	Strongly Agree	Agree	Neither agree or disagree	Disagree	Strongly Disagree
Please select:	○	○	○	○	○

***36. I felt I was in control of my social interactions**

	Strongly Agree	Agree	Neither agree or disagree	Disagree	Strongly Disagree
Please select:	○	○	○	○	○

***37. I preferred to be completely anonymous to others**

	Strongly Agree	Agree	Neither agree or disagree	Disagree	Strongly Disagree
Please select:	○	○	○	○	○

***38. I valued being able to share and exchange information with others**

	Strongly Agree	Agree	Neither agree or disagree	Disagree	Strongly Disagree
Please select:	○	○	○	○	○

***39. It was a good platform for making me feel less isolated and on my own as I manage my pain**

	Strongly Agree	Agree	Neither agree or disagree	Disagree	Strongly disagree
Please select:	○	○	○	○	○

***40. I valued that I was able to connect with people or resources outside of where I live (i.e. in another town, state or country)**

	Strongly Agree	Agree	Neither agree or disagree	Disagree	Strongly Disagree
Please select:	○	○	○	○	○

***41. It was good for searching for and finding useful information**

	Strongly Agree	Agree	Neither agree or disagree	Disagree	Strongly Disagree
Please select:	○	○	○	○	○

***42. It was useful for helping me learn about managing my condition or pain**

	Strongly Agree	Agree	Neither agree or disagree	Disagree	Strongly Disagree
Please select:	○	○	○	○	○

***43. I preferred it if use was facilitated or moderated to maintain the quality of the information**

	Strongly Agree	Agree	Neither agree or disagree	Disagree	Strongly Disagree
Please select:	◯	◯	◯	◯	◯

***44. It helped knowing there are others out there who have shared the same experiences as me**

	Strongly Agree	Agree	Neither agree or disagree	Disagree	Strongly Disagree
Please select:	◯	◯	◯	◯	◯

***45. I was able to learn from finding out about others' experiences**

	Strongly Agree	Agree	Neither agree or disagree	Disagree	Strongly Disagree
Please select:	◯	◯	◯	◯	◯

***46. I valued being able to share my own experiences with others**

	Strongly Agree	Agree	Neither agree or disagree	Disagree	Strongly Disagree
Please select:	◯	◯	◯	◯	◯

***47. My motivation for visiting/using it changed depending on my management needs at particular points in time**

	Strongly Agree	Agree	Neither agree or disagree	Disagree	Strongly Disagree
Please select:	◯	◯	◯	◯	◯

***48. How often I visited/used it changed depending on my management needs at particular points in time**

	Strongly Agree	Agree	Neither agree or disagree	Disagree	Strongly Disagree
Please select:	◯	◯	◯	◯	◯

***49. The specific things I did when I visited/used it changed depending on my management needs at particular points in time**

	Strongly Agree	Agree	Neither agree or disagree	Disagree	Strongly Disagree
Please select:	◯	◯	◯	◯	◯

Facebook Final Question

50. Please use this space if you have any further comments about using FACEBOOK for your pain management..

Follow-up - **PAIN HEALTH YouTube Videos**

The following questions relate specifically to **PAIN HEALTH YouTube videos**

51. Please comment if you particularly LIKED anything about using YOUTUBE as part of your pain management in this study (i.e. features, activities, information, uses, etc)

52. Please comment if you particularly DISLIKED anything about using YOUTUBE as part of your pain management in this study (i.e. features, activities, information, uses, etc)

***53. Did your use of PAIN HEALTH on YouTube lead you to any other online resources? (e.g. other videos, social network sites, blogs, videos, wikis, websites)**

- Yes
- No

***54. If 'yes' to the previous question, please briefly comment on what other types of online resources you came across from using YOUTUBE**

***55. How often did you visit the PAIN HEALTH videos on YouTube during this study?**

- At least daily
- At least weekly
- At least monthly
- Less often
- Never

***56. What YOUTUBE activities did you do? (you can select more than one):**

- ☐ Watch videos
- ☐ Post my own video
- ☐ Share a video
- ☐ Like/rate a video
- ☐ Comment on a video
- ☐ Subscribe to a channel
- ☐ None of the above

Other (please specify)

Thinking about the YOUTUBE activities that you did, please answer the following statements..

***57. I was conscious to control the amount and sorts of things that other people knew about me**

	Strongly Agree	Agree	Neither agree or disagree	Disagree	Strongly Disagree
Please select:	○	○	○	○	○

***58. I felt I was in control of my social interactions**

	Strongly Agree	Agree	Neither agree or disagree	Disagree	Strongly Disagree
Please select:	○	○	○	○	○

***59. I preferred to be completely anonymous to others**

	Strongly Agree	Agree	Neither agree or disagree	Disagree	Strongly Disagree
Please select:	○	○	○	○	○

***60. I valued being able to share and exchange information with others**

	Strongly Agree	Agree	Neither agree or disagree	Disagree	Strongly Disagree
Please select:	○	○	○	○	○

***61. It was a good platform for making me feel less isolated and on my own as I manage my pain**

	Strongly Agree	Agree	Neither agree or disagree	Disagree	Strongly disagree
Please select:	○	○	○	○	○

***62. I valued that I was able to connect with people or resources outside of where I live (i.e. in another town, state or country)**

	Strongly Agree	Agree	Neither agree or disagree	Disagree	Strongly Disagree
Please select:	○	○	○	○	○

***63. It was good for searching for and finding useful information**

	Strongly Agree	Agree	Neither agree or disagree	Disagree	Strongly Disagree
Please select:	○	○	○	○	○

***64. It was useful for helping me learn about managing my condition or pain**

	Strongly Agree	Agree	Neither agree or disagree	Disagree	Strongly Disagree
Please select:	○	○	○	○	○

***65. I preferred it if use was facilitated or moderated to maintain the quality of the information**

	Strongly Agree	Agree	Neither agree or disagree	Disagree	Strongly Disagree
Please select:	◯	◯	◯	◯	◯

***66. It helped knowing there are others out there who have shared the same experiences as me**

	Strongly Agree	Agree	Neither agree or disagree	Disagree	Strongly Disagree
Please select:	◯	◯	◯	◯	◯

***67. I was able to learn from finding out about others' experiences**

	Strongly Agree	Agree	Neither agree or disagree	Disagree	Strongly Disagree
Please select:	◯	◯	◯	◯	◯

***68. I valued being able to share my own experiences with others**

	Strongly Agree	Agree	Neither agree or disagree	Disagree	Strongly Disagree
Please select:	◯	◯	◯	◯	◯

***69. My motivation for visiting/using it changed depending on my management needs at particular points in time**

	Strongly Agree	Agree	Neither agree or disagree	Disagree	Strongly Disagree
Please select:	◯	◯	◯	◯	◯

***70. How often I visited/used it changed depending on my management needs at particular points in time**

	Strongly Agree	Agree	Neither agree or disagree	Disagree	Strongly Disagree
Please select:	◯	◯	◯	◯	◯

***71. The specific things I did when I visited/used it changed depending on my management needs at particular points in time**

	Strongly Agree	Agree	Neither agree or disagree	Disagree	Strongly Disagree
Please select:	◯	◯	◯	◯	◯

YouTube Final Question

72. Please use this space if you have any further comments about using YOUTUBE for your pain management..

Follow-up - **BLOGGING RESOURCES**

The following questions relate specifically to **the various BLOGGING resources supplied**

73. Please comment if you particularly LIKED anything about using BLOGS as part of your pain management in this study (i.e. features, activities, information, uses, etc)

74. Please comment if you particularly DISLIKED anything about using BLOGS as part of your pain management in this study (i.e. features, activities, information, uses, etc)

***75. Did the BLOGS lead you to any other online resources? (e.g. other blogs, social network sites, videos, wikis, websites)**

- Yes
- No

***76. If 'yes' to the previous question, please briefly comment on what other types of online resources you came across from using the BLOGS**

***77. How often did you visit the BLOGS during this study?**

- At least daily
- At least weekly
- At least monthly
- Less often
- Never

***78. What BLOGGING activities did you do? (you can select more than one):**

- [ ] Read a blog
- [ ] Created and posted my own blog
- [ ] Commented on a blog post
- [ ] Share a blog post
- [ ] Subscribe to a blog
- [ ] None of the above

Other (please specify)

Thinking about the BLOGGING activities that you did, please answer the following statements..

***79. I was conscious to control the amount and sorts of things that other people knew about me**

	Strongly Agree	Agree	Neither agree or disagree	Disagree	Strongly Disagree
Please select:	○	○	○	○	○

***80. I felt I was in control of my social interactions**

	Strongly Agree	Agree	Neither agree or disagree	Disagree	Strongly Disagree
Please select:	○	○	○	○	○

***81. I preferred to be completely anonymous to others**

	Strongly Agree	Agree	Neither agree or disagree	Disagree	Strongly Disagree
Please select:	○	○	○	○	○

***82. I valued being able to share and exchange information with others**

	Strongly Agree	Agree	Neither agree or disagree	Disagree	Strongly Disagree
Please select:	○	○	○	○	○

***83. They were a good platform for making me feel less isolated and on my own as I manage my pain**

	Strongly Agree	Agree	Neither agree or disagree	Disagree	Strongly disagree
Please select:	○	○	○	○	○

***84. I valued that I was able to connect with people or resources outside of where I live (i.e. in another town, state or country)**

	Strongly Agree	Agree	Neither agree or disagree	Disagree	Strongly Disagree
Please select:	○	○	○	○	○

***85. They were good for searching for and finding useful information**

	Strongly Agree	Agree	Neither agree or disagree	Disagree	Strongly Disagree
Please select:	○	○	○	○	○

***86. They were useful for helping me learn about managing my condition or pain**

	Strongly Agree	Agree	Neither agree or disagree	Disagree	Strongly Disagree
Please select:	○	○	○	○	○

***87. I preferred it if use was facilitated or moderated to maintain the quality of the information**

	Strongly Agree	Agree	Neither agree or disagree	Disagree	Strongly Disagree
Please select:	◯	◯	◯	◯	◯

***88. It helped knowing there are others out there who have shared the same experiences as me**

	Strongly Agree	Agree	Neither agree or disagree	Disagree	Strongly Disagree
Please select:	◯	◯	◯	◯	◯

***89. I was able to learn from finding out about others' experiences**

	Strongly Agree	Agree	Neither agree or disagree	Disagree	Strongly Disagree
Please select:	◯	◯	◯	◯	◯

***90. I valued being able to share my own experiences with others**

	Strongly Agree	Agree	Neither agree or disagree	Disagree	Strongly Disagree
Please select:	◯	◯	◯	◯	◯

***91. My motivation for visiting/using them changed depending on my management needs at particular points in time**

	Strongly Agree	Agree	Neither agree or disagree	Disagree	Strongly Disagree
Please select:	◯	◯	◯	◯	◯

***92. How frequently I visited/used them changed depending on my management needs at particular points in time**

	Strongly Agree	Agree	Neither agree or disagree	Disagree	Strongly Disagree
Please select:	◯	◯	◯	◯	◯

***93. The specific things I did when I visited/used them changed depending on my management needs at particular points in time**

	Strongly Agree	Agree	Neither agree or disagree	Disagree	Strongly Disagree
Please select:	◯	◯	◯	◯	◯

Blogs Final Question

94. Please use this space if you have any further comments about using BLOGS for your pain management..

END OF QUESTIONS

Thank you for your responses. You have completed the after-study questionnaire and this project. We appreciate you taking the time to be involved. Your responses will be of great insight
