# Supplementary material for: Using Social Media While Waiting in Pain: A Clinical 12-Week Longitudinal Pilot Study
Source: JMIR Res Protoc. 2015 Aug 7;4(3):e101. doi: 10.2196/resprot.4621 (PMC4705018; doi:10.2196/resprot.4621)
Supplement: Multimedia Appendix 6 [file resprot_v4i3e101_app6.pdf]

Appendix 6: Semi-structured Interview Template

How have you been? Which of the resources have you used?

Have you used them for....describe (re: themes and descriptive language according to affordances) – let patients expand

- Exploration (searching):
- Connection (with people)
- Narration (shared experiences)
- Adaptation (varied use, motivation, etc)
- Self-presentation (control, identification)

Any positive or negative sentiments about their use on health outcomes?
