# Supplementary material for: Using Social Media While Waiting in Pain: A Clinical 12-Week Longitudinal Pilot Study
Source: JMIR Res Protoc. 2015 Aug 7;4(3):e101. doi: 10.2196/resprot.4621 (PMC4705018; doi:10.2196/resprot.4621)
Supplement: Multimedia Appendix 7 [file resprot_v4i3e101_app7.pdf]

Appendix 7: Social Media in Chronic Pain Recruitment List

Number	Sex	Age	Diagnosis	Referral Month	Email Sent? y/n	Internet Competence (y/n)	Notes/Comments
SM001	F	65	Trigeminal neuralgia	Dec-13			No Answer/Unreachable
SM002	M	57	LBP/sciatica	Dec-13			No Devices
SM003	F	60		Dec-13			No Answer/Unreachable
SM004	M	34	Headache	Dec-13			Medically Inappropriate
SM005	F	62		Dec-13			No Answer/Unreachable
SM006	F	62	LBP	Jan - April 14			NESB
SM007	F	47	LBP	Jan - April 14			Doesn’t like the Internet and Facebook
SM008	F	61	UL	Jan - April 14			NESB
SM009	M	36	LBP	Jan - April 14			No Devices
SM010	M	26	LBP/social anxiety	Jan - April 14			No Answer/Unreachable
SM011	M	50	UL/LL	Jan - April 14			No Answer/Unreachable
SM012	F	25	UL/LL	Jan - April 14	y		Wanted more info but didn't register or reply
SM013	F	30	LL	Jan - April 14			No Answer/Unreachable
SM014	F	49	Headache	Jan - April 14	y		Prolonged Computer Devices Flares Pain
SM015	F	40	Fibromyalgia	Jan - April 14			Time Poor
SM016	M	49	Neck Pain	Jan - April 14			No Answer/Unreachable
SM017	M	53	LBP	Jan - April 14			No Answer/Unreachable
SM018	F	27	LL	Jan - April 14			No Answer/Unreachable
SM019	F	25	LL/Neck	Jan - April 14	y	y	Wanted more info but didn't register or reply
SM020	F	45	LBP	Jan - April 14	y		Wanted more info but didn't register or reply
SM021	F	35	Head/Neck Pain	Jan - April 14	y	y	Wanted more info - registered and enrolled
SM022	M	60	Pancreatitis	Jan - April 14			No Answer/Unreachable
SM023	F	43	LL	Jan - April 14	y		Medically Inappropriate
SM024	F	49	Neck/LBP	Jan - April 14	y	y	Wanted more info - registered and enrolled
SM025	M	59	LL	Jan - April 14			Moved to Priority
SM026	F	43	LBP	Jan - April 14			No Answer/Unreachable
SM027	F	47	LBP	Jan - April 14			Moved to Priority
SM028	F	36	LBP	Jan - April 14		y	Wanted more info - registered and enrolled
SM029	M	30	LBP	Jan - April 14	y	y	Wanted more info - registered and enrolled
SM030	F	42	LBP	Jan - April 14			Not Interested in Participating
SM031	M	21	LBP	Jan - April 14	y	y	Wanted more info - registered and enrolled
SM032	F	26	Headache	Jan - April 14			Discharged From Wait List
SM033	M	20	Headache	Jan - April 14			Discharged From Wait List
SM034	F	23	UL/LL	Jan - April 14	y	y	Wanted more info - registered and enrolled
SM035	M	58	LBP	Jan - April 14			No Answer/Unreachable
SM036	M	35	LL	Jan - April 14	y		No Answer/Unreachable
SM037	M	23	Thoracic Pain	Jan - April 14	y		No Answer/Unreachable
SM038	F	39	LBP	Jan - April 14	y		No Answer/Unreachable
SM039	F	30	LBP	Jan - April 14			Medically Inappropriate
SM040	F	41	LBP	Jan - April 14			No Answer/Unreachable
SM041	M	44	Widespread Pain	Jan - April 14			Medically Inappropriate
SM042	M	48	Neck Pain	Jan - April 14	y	y	Wanted more info but didn't register or reply
SM043	M	45	LBP	Jan - April 14			No Answer/Unreachable
SM044	M	45	LBP	Jan - April 14			No Answer/Unreachable
SM045	M	32	LBP	Jan - April 14	y	y	Wanted more info but didn't register or reply
SM046	F	27	LBP	Jan - April 14			No Answer/Unreachable
SM047	F	52	LBP	Jan - April 14		n	Time Poor
SM048	F	42	LBP	Jan - April 14			No Answer/Unreachable
SM049	F	37	Widespread pain	Jan - April 14			NESB
SM050	F	35	Head/Neck Pain	Jan - April 14	y	y	Duplicate - Already in System
SM051	F	58	Wide Spread Pain	May-14			No Answer/Unreachable
SM052	F	43	LBP	Jun-14			No Answer/Unreachable
SM053	F	63		Jun-14			No Answer/Unreachable
SM054	M	75	LBP	Jun-14			No Answer/Unreachable
SM055	F	35	LL	Jun-14		y	Wanted more info but didn't register or reply

- Unreachable
- Exluded
- Enroled
- Interested but didn't register

SM056	M	47	LBP	Jun-14			Moved to Priority
SM057	M	19	Testicular pain	Jun-14	y	y	Wanted more info - registered and enrolled
SM058	F	42	LBP	Jun-14	y	y	Not Interested in Participating
SM059	M		LL	Jun-14			Moved to Priority
SM060	F		LBP	Jun-14			No Answer/Unreachable
SM061	F		LBP	Jun-14			Wanted more info but didn't register or reply
SM062	M		UL	Jun-14			No Answer/Unreachable
SM063	M			Jun-14			Medically Inappropriate
SM064	F		LBP	Jun-14			No Answer/Unreachable
SM065	F			Jun-14			No Devices
SM066	M		LBP	Jun-14		y	Wanted more info - registered and enrolled
SM067	F	38	LBP	Jun-14			No Answer/Unreachable
SM068	F		LBP	Jun-14		y	Wanted more info - registered and enrolled
SM069	M	58	LBP	Jun-14			No Answer/Unreachable
SM070	F	39	LBP	Jun-14			NESB
SM071	F	60	LBP	Jun-14			No Answer/Unreachable
SM072	M	54	LBP	Jun-14			No Answer/Unreachable
SM073	M	54	Fibromyalgia	Jun-14			No Answer/Unreachable
SM074	F	38	LBP	Jun-14			Duplicate - Already in System
SM075	M	48	Neck Pain	Jun-14			Prolonged Computer Devices Flares Pain
SM076	F	21	LBP	Jun-14		y	Wanted more info but didn't register or reply
SM077	M	36	LBP	Jun-14	y	y	Wanted more info - registered and enrolled
SM078	F	26	Breast Pain	Jun-14			Discharged From Wait List
SM079	F	40	LBP	Jun-14	y	y	Not Interested in Participating
SM080	F	24	LL	Jun-14			No Answer/Unreachable
SM081	F	36	Widespread pain	Jun-14			No Answer/Unreachable
SM082	M	43	LBP	Jun-14			No Answer/Unreachable
SM083	M	44	Headache	Jun-14			Confident With Self-Management
SM084	M	36	Widespread Pain	Jun-14			Not Interested in Participating
SM085	F	47	Widespread pain	Jun-14			No Answer/Unreachable
SM086	M	41		Jun-14			Not Interested in Participating
SM087	F	21	LBP	Jun-14		y	Wanted more info - registered and enrolled
SM088	M	28	LBP	Jun-14	y	y	Wanted more info but didn't register or reply
SM089	M	67	UL	Jun-14			NESB
SM090	M	32	LBP	Jun-14	y	y	Wanted more info - registered and enrolled
SM091	F	32	LBP	Jun-14	y	y	Not Interested in Participating
SM092	F	56	LBP	Jun-14			No Answer/Unreachable
SM093	F	39	LBP	Jun-14			No Answer/Unreachable
SM094	F	40	LBP	Jun-14	y	y	Wanted more info but didn't register or reply
SM095	F	38	LBP	Jun-14			No Devices
SM096	F	72	LBP/ LL	April - May 14			Moved to Priority
SM097	F	69		April - May 14			Moved to Priority
SM098	F	70		April - May 14			Moved to Priority
SM099	F	71	UL/LL	April - May 14			Discharged From Wait List
SM100	F	68	LL	April - May 14			Moved to Priority
SM101	M	72	LBP	April - May 14			No Answer/Unreachable
SM102	F	53	LBP	Jul-14			No Answer/Unreachable
SM103	F	42	Chest Pain	Jul-14			Medically Inappropriate
SM104	M	41	Thoracic Pain	Jul-14			No Answer/Unreachable
SM105	M	66	LBP	Jul-14			No Answer/Unreachable
SM106	F	42	Neck Pain	Jul-14	y	y	Not Interested in Participating
SM107	F	21	LBP	Jul-14			Not Interested in Participating
SM108	F	55	LL	Jul-14			NESB
SM109	M	50	UL	Jul-14			Moved to Priority
SM110	F	37	LBP	Jul-14			Moved to Priority
SM111	F	46	Fibromyalgia	Jul-14	y	y	Wanted more info - registered and enrolled
SM112	F	18	Abdominal Pain	Jul-14	y	y	Wanted more info - registered and enrolled
SM113	F	53	Abdominal Pain	Jul-14			No Answer/Unreachable
SM114	M	54	LBP	Jul-14	y	y	Wanted more info but didn't register or reply

SM115	F	41	Widespread Pain	Jul-14			Medically Inappropriate
SM116	M	36	LBP	Jul-14			Moved to Priority
SM117	F	23	LBP	Jul-14			No Answer/Unreachable
SM118	F	27	Osteoporosis	Jul-14	y	y	Wanted more info - registered and enrolled
SM119	F			Jul-14			No Answer/Unreachable
SM120	F	50	LBP	Jul-14			NESB
SM121	F	63	LBP	Jul-14			Medically Inappropriate
SM122	M	66	Abdo	Jul-14			NESB
SM123	M	55	Thoracic Pain	Jul-14			No Answer/Unreachable
SM124	F	31	LBP	Jul-14			No Answer/Unreachable
SM125	M	54	Headaches	Jul-14			Prolonged Computer Devices Flares Pain
SM126	F	62		Jul-14			Discharged From Wait List
SM127	M	51	LBP	Jul-14			No Answer/Unreachable
SM128	M		UL/LL	Aug-14			No Answer/Unreachable
SM129	F	35		Aug-14			No Answer/Unreachable
SM130	M		LL	Aug-14			No Answer/Unreachable
SM131	F			Aug-14			Moved to Priority
SM132	M		LBP	Aug-14			Not Interested in Participating
SM133	M			Aug-14			No Answer/Unreachable
SM134	M	29		Aug-14	y	y	Wanted more info - registered and enrolled
SM135	F	33		Aug-14	y	y	Wanted more info - registered and enrolled
SM136	F	54	LBP	Aug-14	y	y	Wanted more info but didn't register or reply
SM137	M	40		Aug-14			No Answer/Unreachable
SM138	F	37		Aug-14			No Answer/Unreachable
